# 704. Contemporary *Salmonella* spp. Infections in Houston, TX (2019 and 2020) and Emergence of Cephalosporin Resistance

**DOI:** 10.1093/ofid/ofab466.901

**Published:** 2021-12-04

**Authors:** christy tabarani, Anthony R Flores, Anthony R Flores, Cesar A Arias, Audrey Wanger

**Affiliations:** 1 University of Texas, McGovern Medical School, Houston, Texas; 2 McGovern Medical School, Houston, TX; 3 CARMiG, UTHealth and Center for Infectious Diseases, UTHealth School of Public Health, HOU, TX ; Molecular Genetics and Antimicrobial Resistance Unit and International Center for Microbial Genomics, Universidad El Bosque, BOG, COL, Houston, Texas; 4 University of Texas Health Science Center, University of Texas Health Science Center, Houston, TX

## Abstract

**Background:**

*Salmonella* spp. Infections are a significant cause of morbidity in children in the United States. Contemporary clinical and microbiological characteristics of pediatric *Salmonella* infections in urban cities are not well described.

**Methods:**

We used a retrospective chart review of records (0-18 years of age) from a network of hospitals (n=11) in Houston, TX. Only patients with *Salmonella* spp. isolated from clinical samples in 2019 and 2020 were included. Demographic, clinical, and microbiological data were extracted from the medical record.

**Results:**

A total of 35 pediatric cases of *Salmonella* spp infection were identified over the two-year period. Median age was 1.6 years with over one-third (13/35, 37.1%) under one year (**Table 1**). Nearly half (15/35, 42.9%) of patients required hospitalization with a median length of stay of 2 days. From cases with available clinical data (n=31), most common symptoms were fever (22/31, 71%) and bloody diarrhea (21/31, 67.7%) (**Table 2**). Bacteremia was detected in 17.1% (6/35) of cases (**Table 3**). Exposure history was elicited in 29% (9/31) of cases with foreign travel being most common risk factor (**Table 2**). All speciated isolates were *Salmonella enterica* with the majority (24/29, 82.8%) subspecies *enterica.* Of 24 samples with serotype information, the most common was *infantis* (**Table 3**). A single isolate was resistant to all antibiotics tested except meropenem (**Table 3**) and was recovered from a patient after travel to Pakistan. Nearly half of patients (15/31, 48.4%) received definitive therapy with a third generation cephalosporin antibiotic. Complications were rare and included septic arthritis/osteomyelitis (n=1), UTI (n=3), coagulopathy (n=1), and hepatitis (n=1).

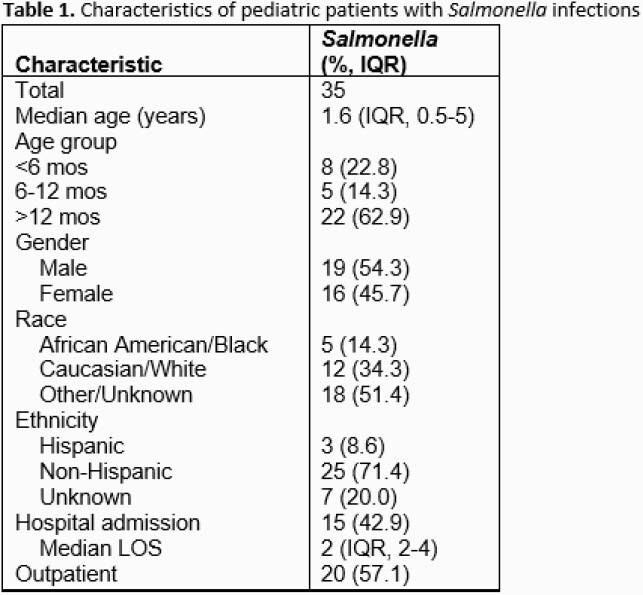

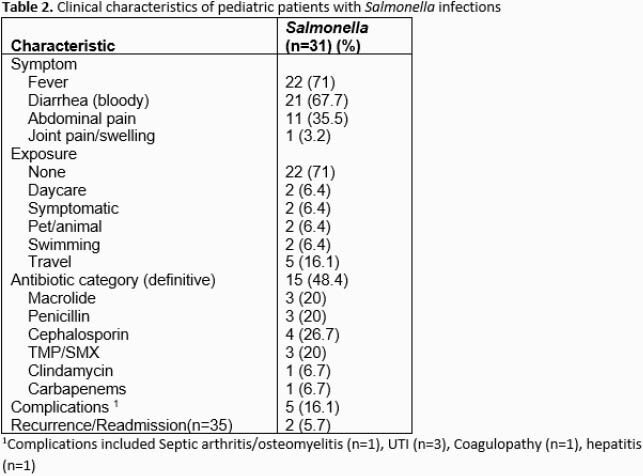

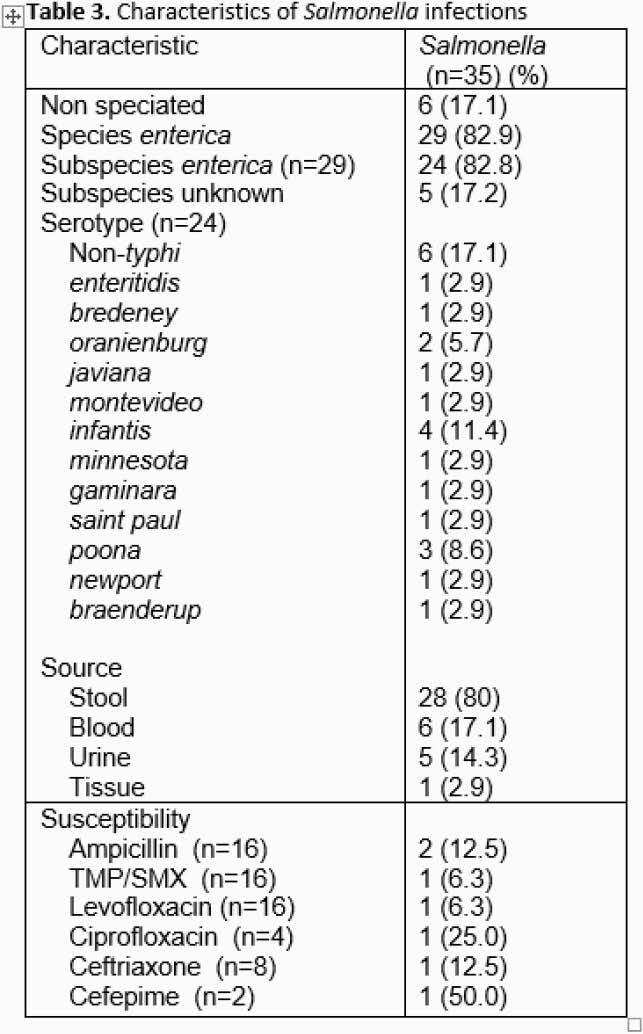

**Conclusion:**

*Salmonella* spp. Infections were common in the Houston metropolitan area over the 2-year period and occurred primarily in young children. Foreign travel seems to be a major risk factor for acquisition of this infection in children. For the first time, the identification of a multi-drug resistant *Salmonella* isolate suggests that this phenotype is likely to increase and highlights the importance of ongoing surveillance.

**Disclosures:**

**Anthony R. Flores, MD, MPH, PhD**, Nothing to disclose **Cesar A. Arias, M.D., MSc, Ph.D., FIDSA**, **Entasis Therapeutics** (Grant/Research Support)**MeMed Diagnostics** (Grant/Research Support)**Merk** (Grant/Research Support)

